# Does COVID-19 vaccination trigger gross hematuria in patients with IgA nephropathy?

**DOI:** 10.1093/ckj/sfae160

**Published:** 2024-05-31

**Authors:** Masahiro Okabe, Nobuo Tsuboi, Saeko Hatanaka, Kotaro Haruhara, Shinya Yokote, Akihiro Shimizu, Takaya Sasaki, Hiroyuki Ueda, Takashi Yokoo

**Affiliations:** Division of Nephrology and Hypertension, Department of Internal Medicine, Jikei University School of Medicine, Tokyo, Japan; Division of Nephrology and Hypertension, Department of Internal Medicine, Jikei University Daisan Hospital, Tokyo, Japan; Division of Nephrology and Hypertension, Department of Internal Medicine, Jikei University School of Medicine, Tokyo, Japan; Division of Nephrology and Hypertension, Department of Internal Medicine, Jikei University Hospital, Tokyo, Japan; Division of Nephrology and Hypertension, Department of Internal Medicine, Jikei University School of Medicine, Tokyo, Japan; Division of Nephrology and Hypertension, Department of Internal Medicine, Jikei University Katsushika Medical Center, Tokyo, Japan; Division of Nephrology and Hypertension, Department of Internal Medicine, Jikei University School of Medicine, Tokyo, Japan; Division of Nephrology and Hypertension, Department of Internal Medicine, Jikei University Kashiwa Hospital, Kashiwa, Japan; Division of Nephrology and Hypertension, Department of Internal Medicine, Jikei University School of Medicine, Tokyo, Japan; Department of Nephrology, Kawaguchi Municipal Medical Center, Kawaguchi, Japan; Division of Nephrology and Hypertension, Department of Internal Medicine, Jikei University School of Medicine, Tokyo, Japan; Division of Nephrology and Hypertension, Department of Internal Medicine, Jikei University Kashiwa Hospital, Kashiwa, Japan; Division of Nephrology and Hypertension, Department of Internal Medicine, Jikei University School of Medicine, Tokyo, Japan; Division of Nephrology and Hypertension, Department of Internal Medicine, Jikei University Hospital, Tokyo, Japan; Division of Nephrology and Hypertension, Department of Internal Medicine, Jikei University School of Medicine, Tokyo, Japan; Division of Nephrology and Hypertension, Department of Internal Medicine, Jikei University Hospital, Tokyo, Japan; Division of Nephrology and Hypertension, Department of Internal Medicine, Jikei University School of Medicine, Tokyo, Japan; Division of Nephrology and Hypertension, Department of Internal Medicine, Jikei University Hospital, Tokyo, Japan

To the Editor,

A number of patients with immunoglobulin A nephropathy (IgAN) have experienced sudden gross hematuria after coronavirus disease 2019 (COVID-19) mRNA vaccination (v-GH) [1]. V-GH typically occurs within days of the second and subsequent vaccination doses, and disappears within a few days, similar to gross hematuria after acute mucosal infection, as part of the natural history of IgAN (n-GH). COVID-19 mRNA vaccination stimulates the production of autoantibodies, including IgA-type [2], analogous to the acute response of mucosal immunity from natural infection. These findings suggest that v-GH and n-GH share a clinical background or immune mechanism, and unraveling these possibilities may provide an opportunity to better understand the pathophysiology of IgAN. Therefore, we aimed to determine the effect of n-GH on the development of v-GH in IgAN patients.

A total of 459 adult IgAN patients with available information regarding n-GH who received COVID-19 mRNA vaccinations were enrolled (Supplemental Methods, Fig. S1, and Table S1). Among the participants, 287 (63%) did not have a history of n-GH, while 172 (37%) did. Of the patients without n-GH and with n-GH, 21 (7.3%) and 18 (10.5%), respectively, had v-GH, with no significant difference between the groups (Fig. 1A). Twelve patients had multiple v-GH, 6 (28.6%) without n-GH and 6 (33.3%) with n-GH, with no significant difference between the groups. The relationship between n-GH and v-GH was similar in subgroup analyses by age and sex (Fig. 1B and C). In another subgroup analysis of IgAN patients who were diagnosed after the initiation of the COVID-19 vaccination campaign, 11 (30.6%) had v-GH in the group without n-GH and 2 (20.0%) had v-GH in the group with n-GH, with no significant difference between the groups (Fig. 1D). Multivariable analysis including previously reported related factors showed no significant association of n-GH with v-GH (Table S2). The incidence of COVID-19 was not associated with that of both v-GH and n-GH (Tables S3 and S4).

**Figure 1:**
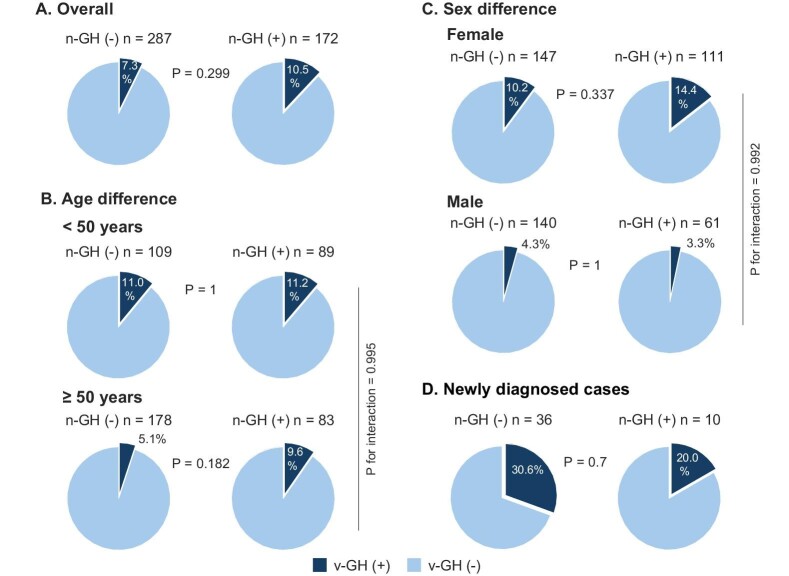
Prevalence of gross hematuria following COVID-19 mRNA vaccination in patients with IgAN in relation to a natural history of gross hematuria. The prevalence of v-GH between patients having IgAN with and without an n-GH (**A**). Subgroup analyses by age group (**B**) and sex (**C**). Prevalence of v-GH in the subgroup of newly diagnosed patients after the initiation of the COVID-19 vaccination campaign (**D**).

The development of n-GH, which is considered an acute exacerbation of IgAN via the activation of mucosal immunity, likely involves increased production of galactose-deficient-IgA1 (Gd-IgA1) and activation of the complementary system, both of which are reported to be stimulated by the COVID-19 mRNA vaccine [3, 4]. Unexpectedly, however, the presence of n-GH in our cohort did not show a clear relationship with the incidence of v-GH, suggesting that the factors governing the sensitivity to v-GH and n-GH are not necessarily identical. In v-GH, there is also the possibility of a unique immune response to COVID-19 mRNA vaccination that precedes the production of Gd-IgA1 or activation of the complement system. For example, an allergic immune response to polyethylene glycol, which is included in the vaccine manufacturing process, may only be involved with v-GH [5]. Another reason for the lack of an association between v-GH and n-GH may be attributed to different immune reactivities due to aging or IgAN treatment.

A major limitation of this study was that the n-GH episodes were dependent on patient reports, and pre-vaccination data for patients with newly diagnosed IgAN triggered by v-GH is lacking. Moreover, this study was mostly conducted on Japanese patients; thus, the results are not necessarily generalizable to other races or geographic regions.

In conclusion, this is the first report to examine the association between v-GH and n-GH in patients with IgAN. Despite some possible similarities in pathogenesis, we failed to demonstrate a clear relationship between v-GH and n-GH, suggesting the involvement of factors specific to each condition. To better understand the pathogenesis of IgAN, further studies are warranted to elucidate the various immunological activation pathways that are commonly or differentially involved in the development of gross hematuria due to infection or vaccination.

## Supplementary Material

sfae160_Supplemental_File

## References

[1] Matsuzaki K, Aoki R, Nihei Y et al. Gross hematuria after SARS-CoV-2 vaccination: questionnaire survey in Japan. Clin Exp Nephrol 2022;26:316–22. 10.1007/s10157-021-02157-x 34773533 PMC8590432

[2] Wisnewski AV, Campillo Luna J, Redlich CA. Human IgG and IgA responses to COVID-19 mRNA vaccines. PLoS ONE 2021;16:e0249499. 10.1371/journal.pone.0249499 34133415 PMC8208542

[3] Arunachalam PS, Scott MKD, Hagan T et al. Systems vaccinology of the BNT162b2 mRNA vaccine in humans. Nature 2021;596:410–6. 10.1038/s41586-021-03791-x. 34252919 PMC8761119

[4] Makita Y, Suzuki H, Kano T et al. TLR9 activation induces aberrant IgA glycosylation via APRIL- and IL-6-mediated pathways in IgA nephropathy. Kidney Int 2020;97:340–9. 10.1016/j.kint.2019.08.022 31748116 PMC7372907

[5] Klimek L, Novak N, Cabanillas B et al. Allergenic components of the mRNA-1273 vaccine for COVID-19: possible involvement of polyethylene glycol and IgG-mediated complement activation. Allergy 2021;76:3307–13. 10.1111/all.14794 33657648 PMC8013891

