# Supplementary material for: Does COVID-19 vaccination trigger gross hematuria in patients with IgA nephropathy?
Source: Clin Kidney J. 2024 May 31;17(7):sfae160. doi: 10.1093/ckj/sfae160 (PMC11217807; doi:10.1093/ckj/sfae160)
Supplement: sfae160_Supplemental_File [file sfae160_supplemental_file.docx]

**Does COVID-19 vaccination trigger gross hematuria in patients with IgA nephropathy?**

Okabe M, Tsuboi N, Hatanaka S, Haruhara K, Yokote S, Shimizu A, Sasaki T, Ueda H, Yokoo T

**Supplementary Data**

**Table of Contents**

- Supplemental Methods
- Supplemental References
- Supplemental Tables
  - Supplemental Table S1
  - Supplemental Table S2
  - Supplemental Table S3
  - Supplemental Table S4
- Supplemental Figures
  - Supplemental Figure S1

**Supplemental Methods**

**Study Design and Patients**

Patients with biopsy-proven immunoglobulin A nephropathy (IgAN) aged >18 years who visited Jikei University Hospital, Jikei Katsushika Medical Center, Jikei Daisan Hospital, or Jikei Kashiwa Hospital between June 1, 2022, and May 31, 2023, were enrolled (**Supplemental Figure S1**). Patients with unvaccinated or unknown coronavirus disease 2019 (COVID-19) mRNA vaccination status; unknown gross hematuria status, history of gross hematuria associated with urolithiasis; insufficient data on the urinalysis and blood test results; complications due to other primary glomerular diseases, kidney or urological cancer, or kidney replacement therapies were excluded. Most patients were included in previously published studies [1,2].

**Clinical Measurements**

Clinical characteristics, including age, sex, height, weight, body mass index, and medical history, including current use of renin-angiotensin-aldosterone system (RAAS) inhibitors, treatment history of corticosteroids or tonsillectomy, and history of gross hematuria, were obtained from the patients’ medical records. Laboratory data from the last outpatient visit before May 31, 2023, and those just before vaccination, including serum creatinine, IgA, C3, urinary protein-to-creatinine ratio (UPCR), and urinary sediments, were also obtained. The estimated glomerular filtration rate was defined using the following formula for Japanese individuals [3]. The red blood cell (RBC) count in the urinary sediment was graded as follows: 0, < 5 RBCs/high-power field (HPF); 1, 5–19 RBCs/HPF; 2, 20–49 RBCs/HPF; and 3, ≥ 50 RBCs/HPF.

**Statistical Analyses**

Continuous variables are presented as medians and interquartile ranges, and categorical variables are expressed as numbers (%). Nonparametric continuous variables were compared using the Mann–Whitney U test for two groups and the Kruskal–Wallis test for more than three groups. Categorical variables were compared using the Fisher’s exact test. The Holm–Bonferroni method was used for the post-hoc analyses. The natural history of gross hematuria and clinically relevant factors, such as age, female sex, and hematuria, were included in the multivariate logistic regression analysis for the incidence of COVID-19 mRNA vaccine-induced gross hematuria. Statistical analyses were performed using R ver. 4.2.2 (The R Foundation for Statistical Computing, Vienna, Austria). Statistical significance was set at P < 0.05.

**Supplemental References**

1. Okabe M, Tsuboi N, Haruhara K, et al. Clinical impact of severe acute respiratory syndrome coronavirus-2 infection on IgA nephropathy. *Nephrology (Carlton)* 2023;28:408–409.
2. Yokote S, Tsuboi N, Shimizu A, et al. Predictors of Gross Hematuria After SARS-CoV-2 mRNA Vaccination in Patients with IgA Nephropathy. *Kidney360* 2023;4:943–950.
3. Matsuo S, Imai E, Horio M. Revised equations for estimated GFR from serum creatinine in Japan. *Am J Kidney Dis* 2009;53:982–992.

**Supplemental Tables**

**Supplemental Table S1. Comparison of the clinical characteristics in all the patients with IgAN included in this study.**

|  | **n-GH (–) group** | | **n-GH (+) group** | | **P-value** | | |
| --- | --- | --- | --- | --- | --- | --- | --- |
|  |  |  |  |  | **Overall** | **Post-hoc analysis** | |
|  | **v-GH (–)**  *n* = 266 | **v-GH (+)**  *n* = 21 | **v-GH (–)**  *n* = 154 | **v-GH (+)**  *n* = 18 |  | **n-GH (–) v-GH (–) group**  vs.  **n-GH (–) v-GH (+) group** | **n-GH (+) v-GH (–) group**  vs.  **n-GH (+) v-GH (+) group** |
| **Age, year** | 55 [44, 66] | 47 [33, 53] | 49 [41, 57] | 45 [34, 53] | < 0.001 | 0.025 | 0.438 |
| **Sex, female** | 132 (49.6) | 15 (71.4) | 95 (61.7) | 16 (88.9) | < 0.001 | 0.208 | 0.1375 |
| **Height, cm** | 163 [155, 171] | 160 [158, 172] | 160 [155, 170] | 158 [156, 167] | 0.435 | ‒ | ‒ |
| **Weight, kg** | 62.0 [54.0, 70.0] | 60.0 [52.6, 65.9] | 57.0 [51.0, 68.0] | 56.0 [48.3, 58.8] | 0.014 | 0.57 | 0.472 |
| **BMI, kg/m^2^** | 22.8 [20.3, 24.9] | 21.7 [20.4, 23.8] | 22.1 [19.9, 24.9] | 20.6 [19.1, 22.9] | 0.067 | ‒ | ‒ |
| **IgAN history, year** | 12 [6, 21] | 3 [2, 6] | 15 [7, 25] | 11 [6, 19] | < 0.001 | < 0.001 | 0.922 |
| **Hypertension** | 162 (60.9) | 9 (42.9) | 68 (44.2) | 4 (22.2) | < 0.001 | 0.337 | 0.332 |
| **Diabetes** | 25 (9.4) | 0 (0.0) | 10 (6.5) | 0 (0.0) | 0.289 | ‒ | ‒ |
| **Current RAAS inhibitors use** | 217 (81.6) | 14 (66.7) | 105 (68.2) | 11 (61.1) | 0.004 | 0.587 | 1 |
| **Past treatment with corticosteroids** | 151 (56.8) | 15 (71.4) | 89 (57.8) | 9 (50.0) | 0.548 | ‒ | ‒ |
| **Past treatment with tonsillectomy** | 110 (41.4) | 14 (66.7) | 82 (53.2) | 9 (50.0) | 0.026 | 0.19 | 1 |
| **Microscopic hematuria** | 60 (22.6) | 10 (47.6) | 45 (29.2) | 16 (88.9) | < 0.001 | 0.048 | < 0.001 |
| **UPCR, g/g** | 0.28 [0.11, 0.57] | 0.25 [0.06, 0.37] | 0.17 [0.08, 0.50] | 0.19 [0.12, 0.45] | 0.237 | ‒ | ‒ |
| **eGFR, mL/min/1.73 m^2^** | 52.1 [38.0, 68.0] | 56.1 [49.0, 76.0] | 59.0 [43.0, 73.0] | 66.5 [56.3, 88.1] | 0.002 | 0.164 | 0.247 |
| **Serum IgA level, mg/dL^†^** | 272 [207, 361] | 208 [169, 322] | 266 [190, 341] | 258 [206, 311] | 0.429 | ‒ | ‒ |
| **Serum C3 level, mg/dL^‡^** | 111 [97, 123] | 110 [93, 120] | 108 [93, 126] | 100 [88, 110] | 0.434 | ‒ | ‒ |

Values are presented as numbers (percentages) or medians [25th to 75th percentiles]. Fisher’s exact test and Kruskal–Wallis test were performed with post-hoc analyses using the Holm–Bonferroni method. ^†^ n = 338; ^‡^ n = 332.

Abbreviations: BMI: Body mass index, eGFR: Estimated glomerular filtration rate; HPF: High-power field; IgAN: IgA nephropathy; n-GH: Natural history of gross hematuria; RAAS: Renin-angiotensin-aldosterone system; UPCR: Urinary protein-to-creatinine ratio; RBC: Red blood cell; v-GH: Vaccination-induced gross hematuria.

**Supplemental Table S2. Relevant factors for the incidence of vaccine-induced gross hematuria in all patients diagnosed with IgAN**

|  | **Univariable** |  | **Multivariable** |
| --- | --- | --- | --- |
|  | **Odds ratio [95% CI]** |  | **Odds ratio [95% CI]** |
| **n-GH** | 1.48 [0.765, 2.860], P = 0.244 |  | 0.891 [0.433, 1.83], P = 0.754 |
| **Female sex** | 3.29 [1.48, 7.34], P = 0.004 |  | 2.79 [1.21, 6.44], P = 0.016 |
| **Age, per year** | 0.960 [0.937, 0.983], P < 0.001 |  | 0.966 [0.943, 0.991], P = 0.007 |
| **Microscopic hematuria** | 6.00 [2.98, 12.1], P < 0.001 |  | 5.26 [2.55, 10.8], P < 0.001 |

The data used in this analysis were the most recently available, as of May 31, 2023.

Abbreviations: IgAN: Immunoglobulin A nephropathy, 95% CI: 95% confidence interval, n-GH: Natural history of gross hematuria.

**Supplemental Table S3. Incidence of COVID-19 in IgA patients with v-GH**

|  | **v-GH (-)** | **v-GH (+)** | **P value** |
| --- | --- | --- | --- |
| **COVID-19 (-)** | 320 (76.4%) | 29 (74.4%) | 0.844 |
| **COVID-19 (+)** | 99 (23.6%) | 10 (25.6%) |  |

COVID-19 incidence was calculated from data until May 31, 2023. Values are presented as numbers (percentages) and Fisher’s exact test was performed.

Abbreviations: v-GH: Gross hematuria after COVID-19 vaccination.

**Supplemental Table S4. Incidence of COVID-19 in IgA patients with n-GH**

|  | **n-GH (-)** | **n-GH (+)** | **P value** |
| --- | --- | --- | --- |
| **COVID-19 (-)** | 221 (77.0%) | 129 (75.0%) | 0.651 |
| **COVID-19 (+)** | 66 (23.0%) | 43 (25.0%) |  |

COVID-19 incidence was calculated from data until May 31, 2023. Values are presented as numbers (percentages) and Fisher’s exact test was performed.

Abbreviations: n-GH: Natural history of gross hematuria.

**Supplemental Figures**

**Supplemental Figure S1. Flowchart of patient selection**

IgAN Patients Attending
Jikei University Hospitals

***n* = 459**

IgAN Patients Attending
Jikei University Hospitals

***n* = 517**

**Exclusions *n* = 58**

No vaccination ***n* = 26**

Unknown vaccination status ***n* = 3**

Unknown gross hematuria status ***n* = 17**

Insufficient data ***n* = 5**

Initiation of kidney replacement therapy ***n* = 3**

Gross hematuria associated with urolithiasis ***n* = 2**

Complication of carcinoma ***n* = 1**

Complication of other glomerular diseases ***n* = 1**
